# PD-1 of *Sigmodon hispidus*: Gene identification, characterization and preliminary evaluation of expression in inactivated RSV vaccine-induced enhanced respiratory disease

**DOI:** 10.1038/s41598-019-48225-x

**Published:** 2019-08-12

**Authors:** Abenaya Muralidharan, Louise Larocque, Marsha Russell, Marybeth Creskey, Changgui Li, Wangxue Chen, Gary Van Domselaar, Jingxin Cao, Terry Cyr, Michael Rosu-Myles, Lisheng Wang, Xuguang Li

**Affiliations:** 1 Centre for Biologics Evaluation, Biologics and Genetic Therapies Directorate, HPFB, Health Canada and WHO Collaborating Center for Standardization and Evaluation of Biologicals, Ottawa, ON Canada; 2 National Institute for Food and Drug Control and WHO Collaborating Center for Standardization and Evaluation of Biologicals, Beijing, China; 3 Department of Biochemistry, Microbiology and Immunology, Faculty of Medicine, University of Ottawa, Ottawa, ON Canada; 4 Human Therapeutics Portfolio, National Research Council of Canada, Ottawa, ON Canada; 5 National Microbiology Laboratory, Public Health Agency of Canada, Winnipeg, MB Canada

**Keywords:** Viral infection, Vaccines

## Abstract

*Sigmodon hispidus* or cotton rat is an excellent animal model for studying human infections of respiratory viruses including respiratory syncytial virus (RSV), which is the leading cause of hospitalization in infants and causes high rates of infection in the elderly and immunocompromised patient populations. Despite several decades of research, no vaccine has been licensed whereas inactivated vaccines have been shown to induce severe adverse reaction in a clinical trial, with other forms of RSV vaccine also found to induce enhanced disease in preclinical animal studies. While arguably the cotton rat is the best small animal model for evaluation of RSV vaccines and antivirals, many important genes of the immune system remain to be isolated. Programmed cell death-1 (PD-1) plays an integral role in regulating many aspects of immunity by inducing suppressive signals. In this study, we report the isolation of mRNA encoding the cotton rat PD-1 (crPD-1) and characterization of the PD-1 protein. crPD-1 bound to its cognate ligand on dendritic cells and effectively suppressed cytokine secretion. Moreover, using the newly acquired gene sequence, we observed a decreased level of crPD-1 levels in cotton rats with enhanced respiratory disease induced by inactivated RSV vaccine, unraveling a new facet of vaccine-induced disease.

## Introduction

Programmed cell death-1 (PD-1) is a receptor that belongs to the CD28 superfamily^1^. It is a type I transmembrane glycoprotein composed of an IgV domain that exists as a monomer on the cell^2^. Upon engagement of one of its two ligands, PD-L1 and PD-L2, it delivers negative signals in the immune system^1^. When induced, PD-1 pathways play crucial roles in regulation of autoimmunity, transplantation immunity, infectious immunity and tumor immunity^1^.

PD-1 expression is tightly regulated. Low basal levels are maintained on resting naïve T cells and some developing thymocytes which creates immune tolerance preventing autoimmunity^3–6^. Following activation, PD-1 is transiently expressed on multiple immune cells such as CD4 and CD8 T cells, B cells, macrophages, natural killer cells and dendritic cells^4,7–13^. High expression is vital for regulatory T cell development while follicular helper T cells constitutively express high PD-1^14–17^.

The role of PD-1 in regulating T cell exhaustion during cancer and chronic infection is well established^6,18^. Specifically, PD-1 expression is upregulated on virus-specific T cells during chronic viral infections^1^. The constant antigen exposure and prolonged T cell receptor stimulation leads to high levels of PD-1 and therefore, T cell exhaustion^19^. Several studies involving a wide range of animal models and virus infections have demonstrated the importance of the PD-1 pathway. In non-human primates, blocking PD-1 resulted in rapid expansion of SIV-specific T cells drastically decreasing plasma viral load^20^. Moreover, in mice, blocking the PD-1 pathway restored cytokine production, increased the number of lymphocytic choriomeningitis virus (LCMV)-specific T cells and enhanced viral clearance^21^. In contrast, blocking PD-1 pathway during a respiratory syncytial virus (RSV) infection in mice enhanced pulmonary inflammation and lung injury with modest effects on viral clearance^22^.

RSV is the leading cause of hospitalization in infants^23–26^ with about 50% of children being infected in their first year of life^27,28^. RSV also causes severe disease in the elderly and immune-compromised patients^23,24,29,30^. In the 1960s, a clinical trial involving formaldehyde-inactivated RSV (FI-RSV) resulted in hospitalization of 80% of the participants and 2 deaths following a RSV infection^31–34^. This severe adverse reaction, commonly known as vaccine-induced enhanced respiratory disease (ERD), is yet to be fully understood but might be linked to the induction of a Th2-biased immune response leading to pulmonary inflammation, airway obstruction and mucus hypersecretion as observed in the trial participants and some animal models^35–38^.

*Sigmodon hispidus* (cotton rats) share many similarities in pathology with humans when infected by RSV. Notably, most findings in relation to FI-RSV induced ERD have been replicated in the cotton rats, making them one of the ideal animal models for RSV infection^39–42^. Indeed, cotton rats have proven very useful in the study of human respiratory virus infections including the development and testing of antiviral drugs and vaccines for RSV, measles, influenza, human parainfluenza and human metapneumovirus^43^. However, one of the major drawbacks with this animal model is the availability of research reagents because the genome has not been fully sequenced. As of today, approximately 300 genes have been sequenced in cotton rats that show 75–95% identity to mice and about 50% to humans^44^, while few genes of the immune system have been sequenced. With increasing number of vaccines and therapeutics being evaluated in cotton rats prior to clinical trials, it would be important to better understand the immune system of this animal model^45–47^.

It has been shown that PD1-PDL1 activation is vital for limiting immunopathology in the context of a primary RSV infection in mice^22,48^. However, there have been no studies on the levels of PD-1 in cotton rats experiencing vaccine-induced ERD. Here, we report the isolation of cotton rat PD-1 (crPD-1) gene and characterization of the putative PD-1 protein. Using the newly identified gene sequence as a probe, we found significantly decreased levels of the PD-1 gene in ERD cotton rats following vaccination with FI-RSV, suggesting that downregulation of PD-1 could be associated with excessive pulmonary inflammation.

## Results

### Identification of cotton rat PD-1 sequence, species alignment and putative domains

**Figure 1:**
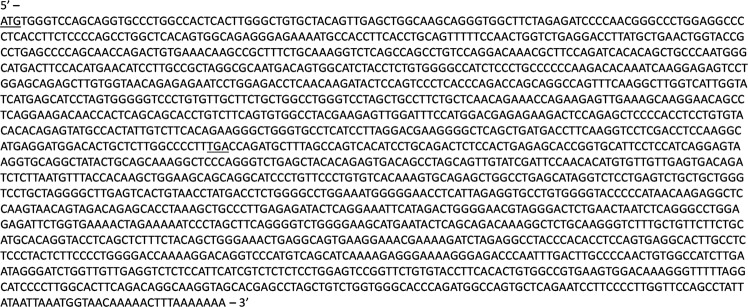
Cotton rat (*Sigmodon hispidus*) PD-1 mRNA sequence. 3′ RACE strategy was used on total RNA extracted from the spleen of a naïve cotton rat to determine the mRNA sequence. The predicted start and stop codon are underlined.

The mRNA sequence of cotton rat programmed cell death receptor-1 (crPD-1) was isolated from cotton rat spleens (Fig. 1). A 3′ RACE strategy was applied, as previously described^49^, using total RNA extracted from spleens as the starting material. Following isolation of mRNA from the total RNA using an oligo dT, primers designed from rodent consensus sequences were used to sequence from the 3′ end to the 5′ end in a stepwise fashion. The ORF was found to be 858 bp in length encoding 285 amino acids (aa) followed by a stop codon and 1027 bp 3′ un-translated region.

**Figure 2:**
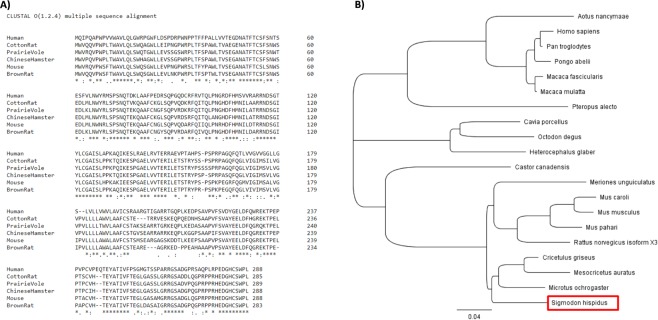
Protein sequence alignment of the cotton rat PD-1. (**A**) Protein sequence of closely related species and human were aligned with crPD-1 using the Clustal Omega tool from EMBL-EBI. Human (*Homo sapiens* NCBI Reference Sequence: AAC51773.1), Prairie Vole (*Microtus ochrogaster* NCBI Reference Sequence: XP_005361412.1), Chinese Hamster (*Cricetulus griseus* NCBI Reference Sequence: XP_003499314.1), Mouse (*Mus musculus* NCBI Reference Sequence: NP_032824.1), and Brown Rat (*Rattus norvegicus* NCBI Reference Sequence: XP_017451871.1). An asterisk (*) indicates positions which have a single, fully conserved residue. A colon (:) indicates conservation between groups of strongly similar properties, scoring >0.5 in the Gonnet PAM 250 matrix. A period (.) indicates conservation between groups of weakly similar properties, scoring = < 0.5 in the Gonnet PAM 250 matrix. (**B**) A phylogenetic tree was produced using Geneious software.

Alignment of the crPD-1 protein sequence with other species revealed an 88% identity with Chinese hamster, 87% with prairie vole, 84% with brown rat, 82% with mouse, and 59% with human PD-1 (Fig. 2A). Phylogenetic tree analysis showed a shared homology of crPD-1 with other members of the Cricetidae family (Fig. 2B).

**Figure 3:**
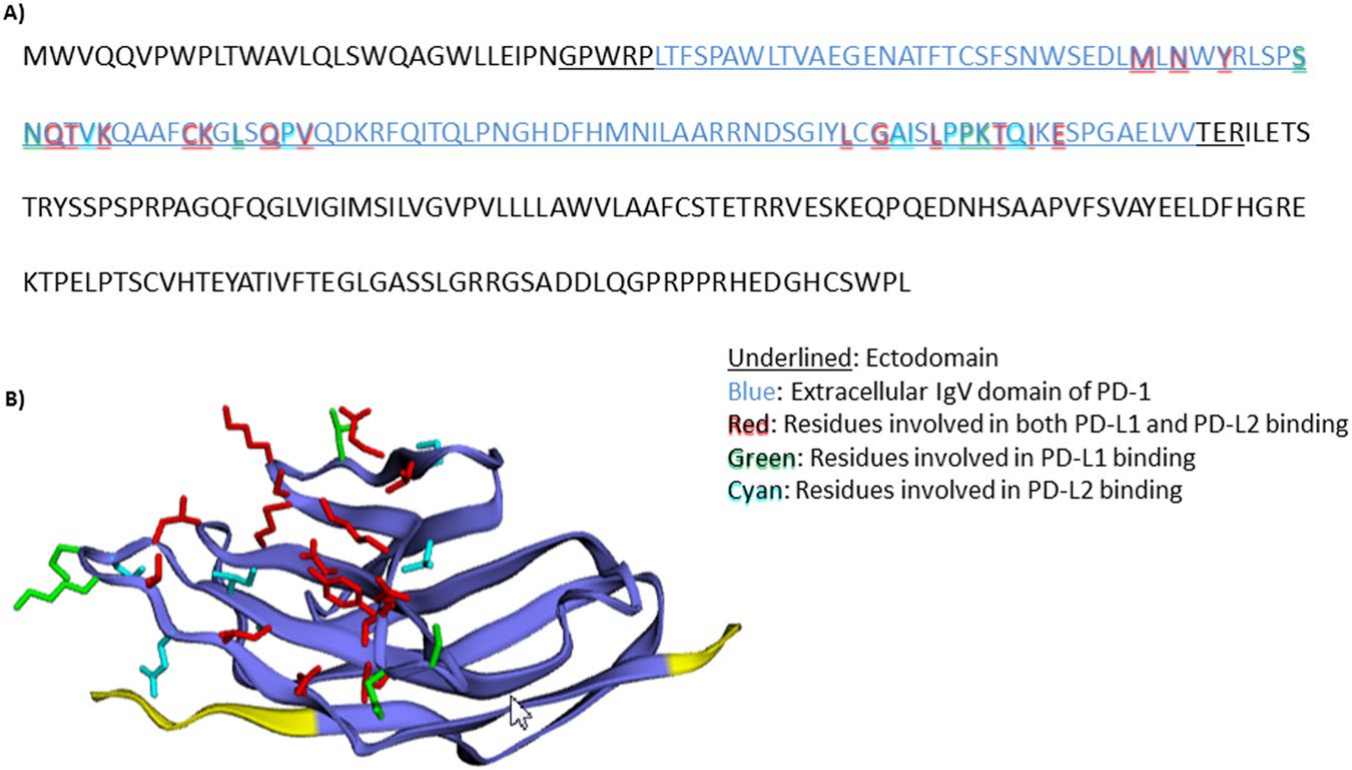
Identification of putative conserved domains in the cotton rat PD-1. (**A**) The underlined sequence indicates the putative ectodomain of PD-1; amino acids in blue are the putative extracellular IgV domain of PD-1; putative residues involved in both PD-L1 and PD-L2 binding are highlighted in red; putative residues involved in PD-L1 binding only are highlighted in green and residues involved in PD-L2 binding only in cyan. (**B**) Predicted structure of the putative ectodomain of the cotton rat PD-1 monomer shown in blue and yellow where the blue region is the putative extracellular IgV domain, residues in red are involved in both PD-L1 and PD-L2 binding, residues in green are involved in PD-L1 binding only and residues in cyan are involved in PD-L2 binding only.

Next, we mapped the structure and functional domains of crPD-1 by comparing to other well-characterized PD-1. In Fig. 3, various putative domains on crPD-1 are annotated. The putative ectodomain spanning amino acid 30 to 147 consists of a single extracellular IgV domain of PD-1 (aa 35–144), which is common to members of the CD28 family^1^. Within the putative IgV domain are the residues involved in the binding of the two ligands of PD-1, PD-L1 and PD-L2. Residues S73, N74, L86, P130 and K131 are involved in PD-L1 binding whereas residues V77, P89, A125, I126, P129 and Q133 bind PD-L2. Amino acids M64, N66, Y68, Q75, T76, K78, C83, K84, Q88, V90, L122, G124, L128, T132, I134 and E136 on PD-1 are involved in both PD-L1 and PD-L2 binding. The structure of the putative ectodomain of crPD-1 was visualized using EZmol software and is shown in Fig. 3B ^50^.

### *In vitro* expression of recombinant cotton rat PD-1

**Figure 4:**
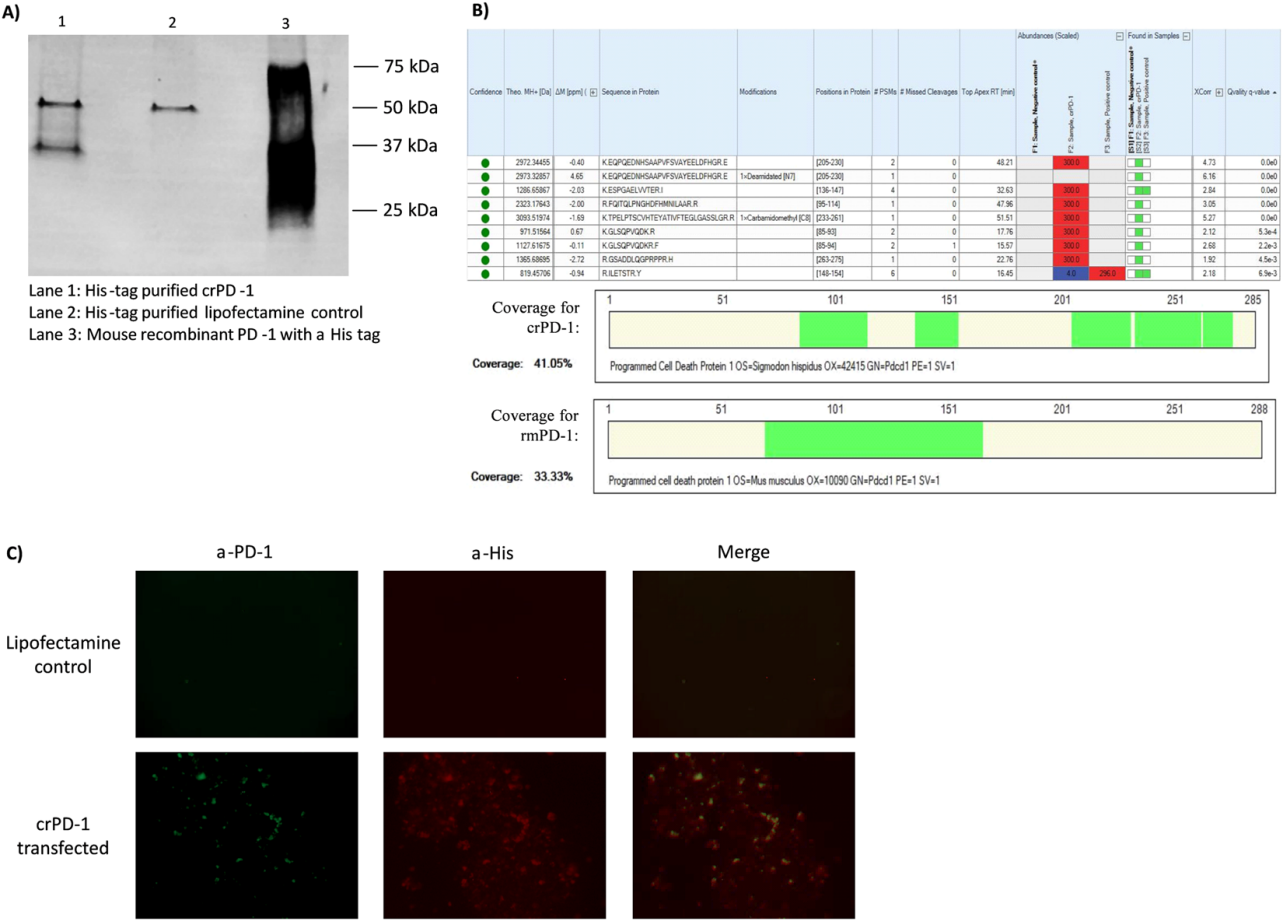
Cotton rat PD-1 protein expression. crPD-1 gene also encoding rat codon optimized secretion signal and ten histidine residues at the 5′-end was synthesized and cloned into pcDNA3.1(+) vector. 293T cells were then transfected for 24 hours, the lysate was collected and his-tag purified. (**A**) Protein expression was confirmed with western blot using a mouse anti-histidine antibody. The expected size of crPD-1 is 36.4 kDa. A His-tag conjugated truncated recombinant mouse PD-1 (rmPD-1) was used as a positive control and, as expected, migrated from 25 to 45 kDa due to different glycosylation and may have aggregates depending on the reducing conditions. A full-length blot is shown as Supplementary Fig. S1. (**B**) Mass spectrometry was performed with his-tag purified lipofectamine control and crPD-1 along with rmPD-1. Seven peptides in the newly found sequence were found in the crPD-1 sample at high abundance and two peptides were found at low abundance with total sequence coverage of 41%. No peptides were found in the lipofectamine control, as expected and two peptides were found in the rmPD-1 sample. Mouse PD-1 sequence coverage for the positive control sample (rmPD-1) was 33.33%. (**C**) Immunofluorescence was also used for protein expression. Cells were permeabilized and stained 24 hours post-transfection. A rabbit anti-mouse PD-1 with Cy2-conjugated anti-rabbit IgG and mouse anti-his tag with Alexa Fluor 555 anti-mouse IgG were used. Representative image of the stained cells at 20X magnification is shown. A merge of the two fluorochromes shows the co-expression of PD-1 and the his-tag, as expected.

For further validation, the sequenced crPD-1 gene was synthesized and cloned into a pcDNA3.1(+) vector. The synthesized gene also consisted of rat codon optimized secretion signal and ten histidine residues at the 5′-end. Expression of this recombinant crPD-1 was conducted in 293T cells. Following His-tag purification, protein expression was confirmed using western blot with anti-His antibody (Fig. 4A), mass spectrometry (Fig. 4B) and immunofluorescence staining of transfected cells with both anti-PD1 and anti-His antibodies (Fig. 4C). Under reducing conditions, the western blot showed a band at 37 kDa for the crPD-1 whereas this band was absent in the lipofectamine control (no plasmid). A band seen at approximately 60 kDa in both crPD-1 and lipofectamine control is likely to be cross-reaction of the antibody with proteins in the media, likely albumin. To serve as a positive control, a mouse recombinant PD-1 (rmPD-1) containing a His-tag was run alongside (Fig. 4A).

Next, mass spectrometry analysis of the purified samples and rmPD-1 against the newly obtained crPD-1 sequence revealed a match of seven peptides in the crPD-1 sample found at high abundance and two peptides at low abundance with total sequence coverage of 41% (Fig. 4B). No peptides from the crPD-1 sequence were found in the lipofectamine control and two peptides were found in the rmPD-1 sample. Comparing the rmPD-1 against the mouse PD-1 sequence resulted in sequence coverage of 33% (Fig. 4B).

Since the commercial anti-human/mouse/rat PD-1 antibody could not detect the rmPD-1 under reducing conditions during western blotting, immunofluorescence was used to confirm co-expression of crPD-1 and the his-tag. While the lipofectamine control showed no fluorescence, crPD-1 transfected 293 T cells showed fluorescence with both antibodies (Fig. 4C). Co-expression is shown by merging the two fluorochromes.

### Characterization of crPD-1 functional activity *in vitro*

**Figure 5:**
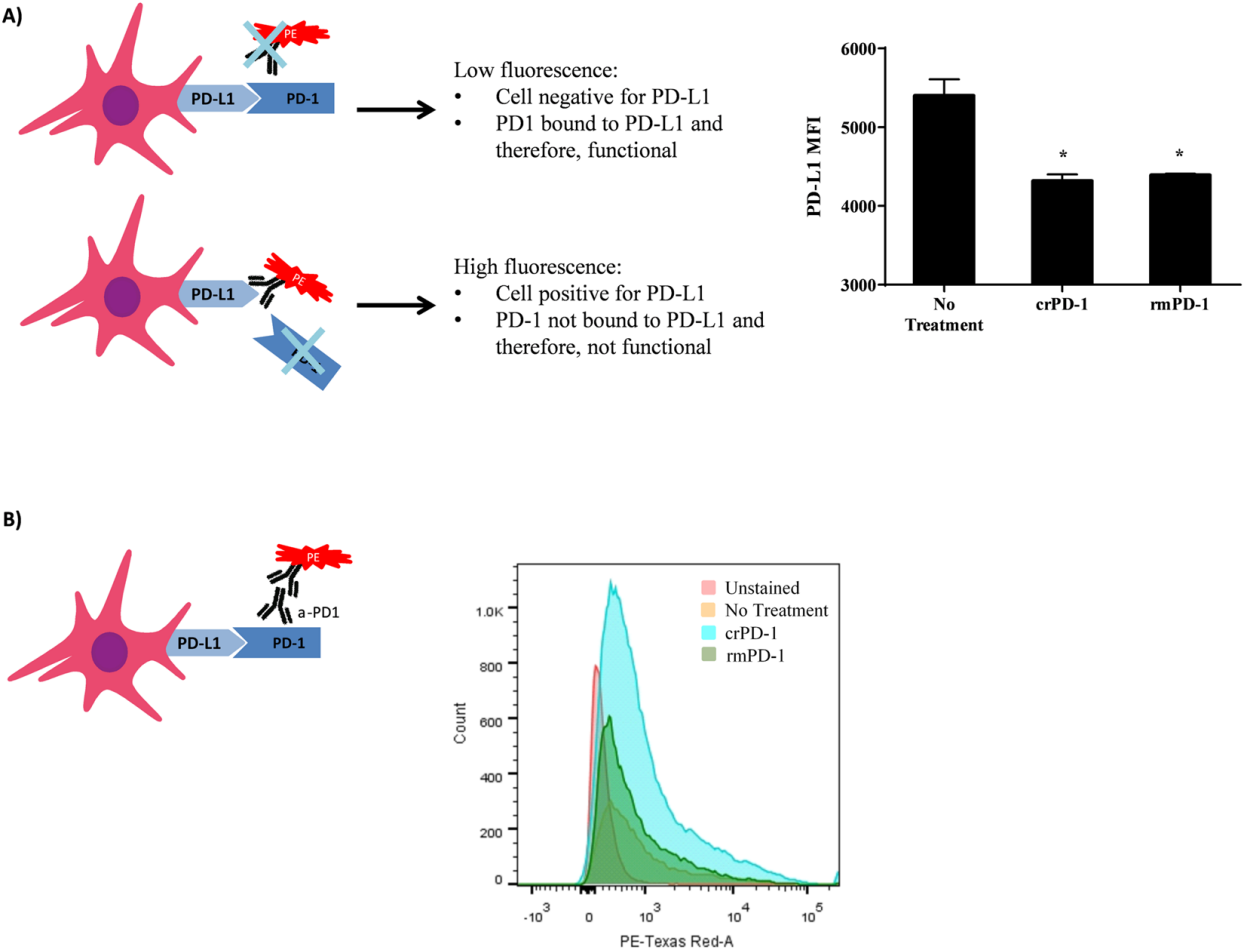
crPD-1 binds PD-L1 on dendritic cells *in vitro*. Purified crPD-1 was added to mouse dendritic cells for 4 hours. Recombinant mouse PD-1 (rmPD-1) and no treatment controls were used. (**A**) The cells were then stained with a fixable viability dye and PE-conjugated anti-human/mouse PD-L1 blocking antibody for flow cytometry analysis. The schematic displays the strategy used (top left). The mean fluorescence intensity (MFI) of PD-L1 among viable cells is shown. Statistical difference between PD-1 treated and no treatment group is indicated. Data shown is mean ± SEM representative of 2 independent experiments; n = 3 per treatment in each experiment; *p < 0.05 (one-way ANOVA with Bonferroni posttest). (**B**) The cells were also stained with rabbit anti-mouse PD-1 primary antibody along with a PE-conjugated anti-rabbit secondary antibody for flow cytometry analysis. The schematic displays the strategy used (bottom left). A histogram of the results representative of 2 independent experiments is shown.

As crPD-1 and mouse PD-1 share 82% protein identity with similar functional domains, we used mouse bone marrow derived dendritic cells (DCs) expressing PD-L1 to evaluate the functional activity of crPD-1. To this end, DCs were incubated with purified crPD-1 or rmPD-1 for 4 hours. Following incubation, the DCs were stained with a viability dye and an anti-human/mouse PD-L1 blocking antibody for flow cytometry analysis. A competitive binding strategy summarized in Fig. 5A (left) was applied, i.e. if the added PD-1 protein was in its functional conformation, it would bind to PD-L1 expressed on the DCs preventing the PD-L1 blocking antibody from binding to the ligand resulting in lower fluorescence than cells not treated with a PD-1 receptor. However, if PD-1 cannot bind PD-L1 or binds with low affinity, the PD-L1 antibody would bind its epitope and, as a result, fluorescence would be quantitatively detected. Our results show that among viable cells, the detected mean fluorescence intensity (MFI) of PD-L1 significantly decreased with the addition of crPD-1 (p = 0.039) and rmPD-1 (p = 0.04) compared to no treatment control (Fig. 5A). Additionally, when detected with an anti-mouse PD-1 antibody, increased number of PD-1 positive dendritic cells were observed after stimulation with rmPD-1 and crPD-1 compared to the untreated control (Fig. 5B). Some positive cells observed in the untreated control reflect the background associated with the PE-conjugated secondary antibody. Furthermore, it is interesting to note the higher positive cell count in the crPD-1 stimulated group compared to rmPD-1 group. This observation is yet to be understood but likely due to the full-length crPD-1 which may have bound stronger to the antibodies used than the rmPD-1, which is a commercially available truncated form of the mouse protein. Nevertheless, these results confirm that the expressed crPD-1 protein is capable of binding to its cognate ligand.

We next investigated whether crPD-1 could reduce IL-6 and TNF-α secretion by activated DCs, given interaction between PD-1 and its ligands is known to induce immunosuppressive pathways leading to decreased pro-inflammatory cytokine production^1,22,51^. To this end, mouse DCs were stimulated with LPS along with crPD-1 or rmPD-1 for 24 hours prior to ELISA quantitation of IL-6 and TNF-α. Compared to the control group treated with LPS only, both crPD-1 and rmPD-1 treatment resulted in considerable decrease of IL-6 and TNF-α expression (Table 1). While magnitude of decreased IL-6 levels was comparable between crPD-1 and rmPD-1, rmPD-1 is slightly more effective in reducing TNF-α expression by mouse DCs, an observation likely due to imperfect match between the two species. Nonetheless, these results indicate that crPD-1 could effective suppress proinflammatory cytokine production, consistent with functional roles played by PD-1 derived from other species including human and mouse.

**Table 1:** crPD-1 downregulates expression of cytokines by dendritic cells *in vitro*.

	IL-6 (pg/ml)		TNF-α (pg/ml)	
	Mean	95% CI	Mean	95% CI
No Treatment	3118	2839 to 3459	3762	3422 to 4128
crPD-1	2636	2400 to 2924	3214	2885 to 3558
rmPD-1	2666	2427 to 2958	3189	2861 to 3532

Mouse dendritic cells were stimulated with LPS along with purified crPD-1 for 24 hours. No treatment control containing LPS only and rmPD-1 control containing LPS and rmPD-1 were used. The supernatant was collected for cytokine quantitation using ELISA. Data shown is representative of 2 independent experiments; n = 3 per treatment in each experiment.

crPD-1: cotton rat PD-1; rmPD-1: recombinant mouse PD-1; CI: confidence interval.

### Downregulation of crPD-1 in ERD cotton rats

**Figure 6:**
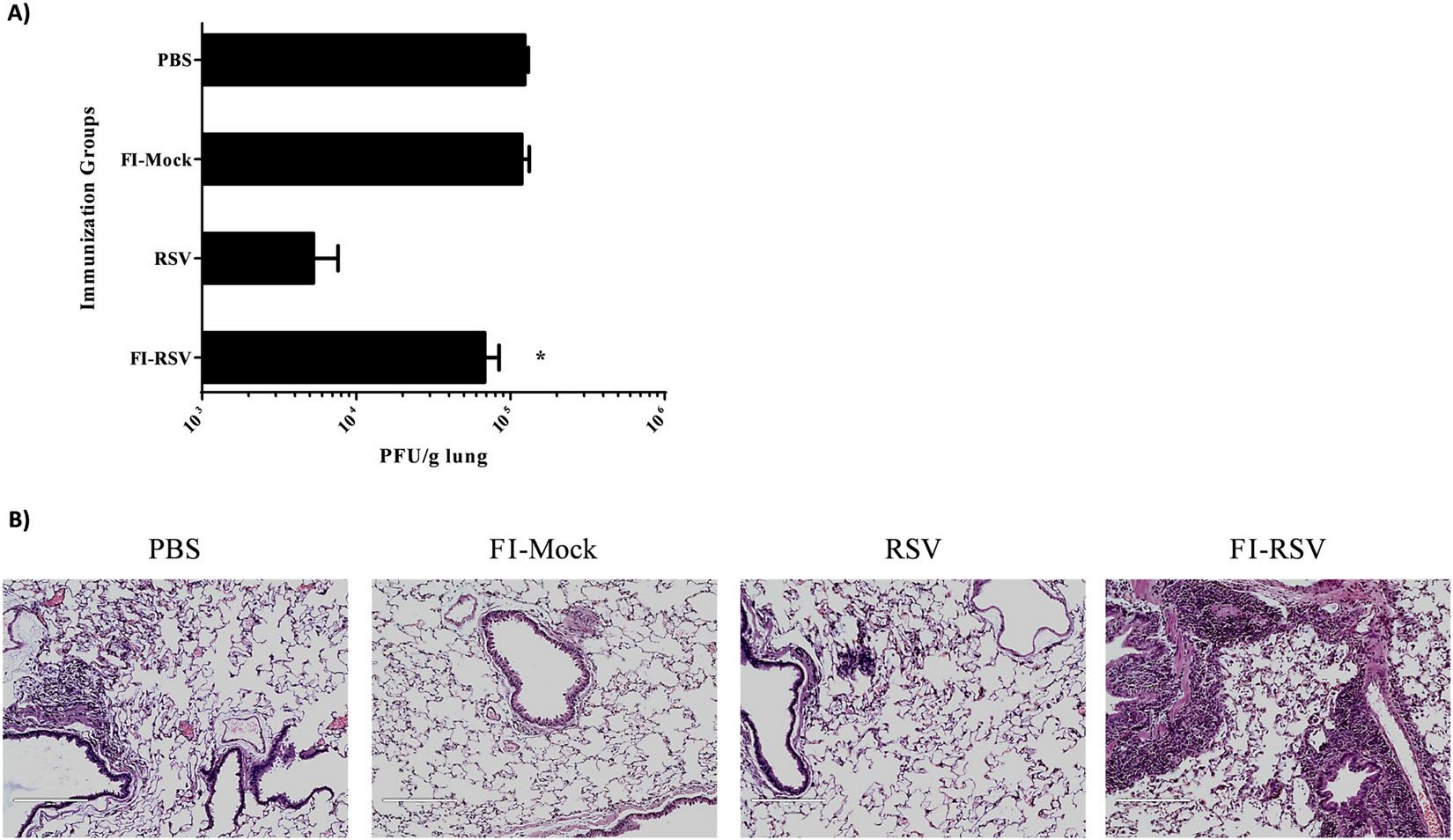
FI-RSV immunization of cotton rats results in ineffective viral clearance with pronounced ERD. Cotton rats were immunized twice 21 days apart with FI-RSV, FI-Mock or PBS intramuscularly or wild-type RSV-A2 intranasally. Four weeks following second immunization, the animals were challenged with RSV-A2 intranasally and euthanized 5 days post-challenge for collection of lungs. (**A**) Lung viral titer determined using plaque assay post challenge. (**B**) Representative images of H&E stained cotton rat lungs post challenge at 20X magnification. Data shown is mean ± SEM representative of 2 independent experiments; n = 3 per group in each experiment; *p < 0.05 (one-way ANOVA with Bonferroni posttest). FI-RSV: Formaldehyde-inactivated RSV; FI-Mock: Formaldehyde-inactivated cell control; PBS: Phosphate-buffered saline.

Having confirmed the functional activity of isolated crPD-1 *in vitro*, we set out to investigate whether crPD-1 could be downregulated in cotton rats suffering enhanced respiratory disease (ERD) as a result of inactivated RSV vaccination, given exacerbated pulmonary inflammation is one of the well-documented pathological findings^37,43^. To this end, we first established the ERD model based on previously reports^36,37,40^. Cotton rats were immunized twice with formaldehyde-inactivated RSV (FI-RSV), formaldehyde-mock control (FI-Mock), live wild-type RSV-A2, or saline (PBS), followed by viral challenge with RSV-A2 four weeks after second immunization. The animals were euthanized 5 days post-challenge for lung viral titer and pathological analysis. Unlike FI-Mock and PBS-immunized rats, immunization with wild-type RSV resulted in effective clearance of virus in the lungs (Fig. 6A); FI-RSV immunization also effectively reduced virus replication albeit less effectively than RSV immunization (p = 0.015).

Next, we examined hematoxylin and eosin (H&E) stained lung tissues post-challenge to confirm the extensive tissue damage that accompanies FI-RSV induced ERD, as observed in previous studies^52^. Prominent alveolitis with infiltrating neutrophils, macrophages and lymphocytes along with a few eosinophils in alveolar spaces were clearly observed in the FI-RSV immunized cotton rats, whereas other immunization groups did not display similar pathological presentations (Fig. 6B). Moreover, peribronchiolitis had a similar pattern of cellular infiltration as alveolitis as well as marked perivascular leukocyte infiltrates throughout the lung sections, which were absent or very mild in other groups (Fig. 6B).

**Figure 7:**
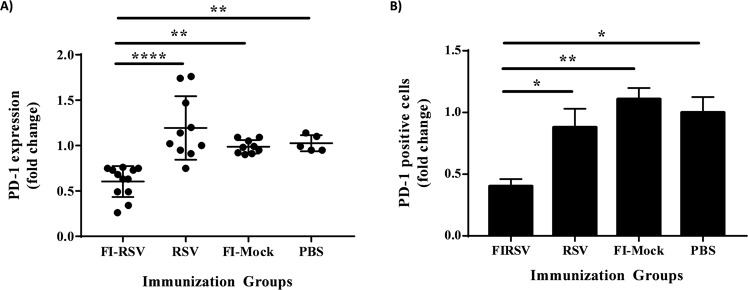
PD-1 gene and protein expression is downregulated in FI-RSV immunized cotton rats experiencing ERD. Lungs were collected from twice-immunized and challenged cotton rats. (**A**) RNA isolated from the lungs were analyzed for PD-1 gene expression using quantitative real-time PCR. C_T_ values were first normalized to β-actin, then, presented as fold change over no immunization control groups, i.e., FI-Mock and PBS that were similar in PD-1 expression levels. (**B**) Charged slides made from lungs fixed in 10% formalin were trimmed, processed and embedded into paraffin blocks were used for immunohistochemistry analysis of PD-1 protein expression using a mouse PD-1 antibody. The percentage of PD-1 positive cells is presented as fold change over no immunization control groups, i.e., FI-Mock and PBS. Data shown is mean ± SEM representative of 2 independent experiments; n = 10 per group in (**A**) and n = 3 per group in (**B**); *p < 0.05, **p < 0.01, ****p < 0.0001 (one-way ANOVA with Bonferroni posttest). FI-RSV: Formaldehyde-inactivated RSV; FI-Mock: Formaldehyde-inactivated cell control; PBS: Phosphate-buffered saline.

Having observed pathological presentations of ERD in FI-RSV immunized cotton rats, we next investigated the PD-1 mRNA expression in the lung tissue using primers and probes designed from the newly acquired gene sequence. Using quantitative real-time PCR, C_T_ values were collected and first, normalized to β-actin for each cotton rat, then, analyzed for fold change over unimmunized controls, FI-Mock and PBS that showed the same levels of PD-1 expression. FI-RSV immunized rats showed a 50% decrease in pulmonary PD-1 expression compared to FI-Mock, whereas RSV immunized rats had a fold change of 1 (Fig. 7A). Moreover, FI-RSV induced a significant downregulation of PD-1 in the lung tissues compared to live RSV immunization (p < 0.0001). Moreover, for evaluation of PD-1 protein expression, lungs from immunized cotton rats collected 5 days post challenge were formalin-fixed and paraffin embedded for immunohistochemistry analysis using a mouse PD-1 antibody (Fig. 7B). Significant reduction in the percentage of PD-1 positive cells, approximately 50%, was also observed at the protein level in FI-RSV immunized cotton rats compared to RSV (p = 0.0379), FI-Mock (p = 0.0058) and PBS (p = 0.0123) immunized cotton rats. Taken together, these results revealed that decreased levels of pulmonary PD-1 at the mRNA and protein level was associated with ERD in animals vaccinated with inactivated RSV vaccine upon subsequent viral infection.

## Discussion

*Sigmodon hispidus* or cotton rats are an excellent animal model for studying human respiratory virus infections^37,43^. For RSV infections, cotton rats are considered the gold standard for development of antivirals, vaccines and biotherapeutics^45–47^. While this animal model was successfully used to determine the dosing, safety and efficacy of the only licensed therapeutic antibody against RSV^45–47^, very few genes of cotton rats have been cloned and characterized, substantially limiting this model’s wider applications, particularly for mechanistic investigation of virus-induced pathogenesis and immune responses. Here, we report, for the first time, the mRNA sequence of cotton rat PD-1 and its expression in inactivated RSV vaccine-induced ERD.

Similar to other genes sequenced in the cotton rats^44^, crPD-1 shares 75–95% homology to mice and rats, and about 50% homology to humans (Fig. 2); phylogenetic analysis showed higher amino acid identity to its own family, Cricetidae, and distant relationship to primates, as expected. Moreover, structural mapping of the putative functional domains revealed high conservation rates among rodents, especially for residues involved in PD-L1 and PD-L2 binding (Fig. 3).

Recombinant crPD-1 protein produced by cells transfected with the newly-isolated gene was found to effectively bind PD-L1 (Fig. 5) on DCs; it could also suppress cytokine production by activated DCs (Table 1), confirming the functional activity of crPD-1. Given the increasing popularity of the cotton rat model for the study of respiratory infection and immunological regulation, in depth characterization of crPD-1 biological activities should be conducted in comparison with PD-1 derived from other animal species and humans, which is ongoing in our laboratory.

With the newly isolated crPD-1 gene as a molecular probe, we determined the expression of crPD-1 in the lungs of wild-type RSV and FI-RSV immunized cotton rats following RSV challenge. As previously reported^36,52^, cotton rats twice-vaccinated and challenged with live RSV were able to effectively clear the virus from their lungs and had mild pulmonary inflammation accompanied by cellular infiltration, while FI-RSV immunized rats had moderate viral clearance and exacerbated pulmonary inflammation (Fig. 6). Importantly, we found significant downregulation of PD-1 in the FI-RSV group at the mRNA and protein level, whereas the level of PD-1 remained unchanged in other groups including live RSV vaccination and primary infection controls, PBS and FI-Mock (Fig. 7). Indeed, increased production of proinflammatory cytokines is one of several significant immunological deregulations in ERD cotton rats vaccinated with inactivated RSV followed by virus infection^53–55^. Taken together, these observations indicate that PD-1, while not implicated in viral clearance, may have significantly contributed to exaggerated pulmonary pathology and ERD. It is of note that the mRNA and protein levels were analyzed five days post RSV challenge, given this time point is the peak of infection in cotton rats. Future studies should be considered to evaluate the mRNA and protein levels at different time points post challenge as reported by other investigators studying the relationship between PD-1 expression and RSV infection in mice^22^.

While this is the first report of PD-1 expression levels evaluated in the context of RSV vaccine-induced ERD, the relationship of reduced levels of PD-1 and enhanced pulmonary pathology could also be corroborated by findings from the mouse model of severe RSV infection. Specifically, following high dose of RSV infection, exacerbated pulmonary inflammatory response was characterized with over production of pro-inflammatory cytokines and increased infiltration of inflammatory cells similar to that of vaccine-induced ERD^56–58^, while blockade of PD1-PD-L1 pathways at the time of T cell infiltration into the lungs resulted in augmentation of pulmonary inflammation and tissue injury with minimal effects on viral clearance^22^. Indeed, the engagement of PD-1 with inflammatory DC-derived PD-L1 is crucial for regulation of pro-inflammatory cytokine release by effector CD4 and CD8 T cells, resulting in control of effector T cell activities in the lungs. In addition, they showed that blocking PD-L1 following RSV infection enhanced weight loss and lung histopathology in mice^22,59^. Taken together, our observations are in good agreement with severe RSV infection in the murine model where low levels of PD-1 induction accompany enhanced respiratory disease.

In short, the gene encoding crPD-1 is 858 bp in length encoding 285 amino acids followed by a stop codon and 1027 bp 3′ un-translated region. The protein shares homology of 82–88% with other small rodents and 59% with its human counterpart. Functional characterization revealed that the crPD-1 protein bound to its ligand expressed on dendritic cells and effectively suppressed IL-6 and TNF-α secretion. Moreover, PD-1 gene expression was substantially downregulated in the lung tissues of the cotton rats with ERD, suggesting its possible involvement in exacerbated pulmonary inflammation in the diseased animals. The availability of the PD-1 gene and protein could facilitate future studies of vaccine-induced protection or -associated disease enhancement in addition to other immunological investigations in the cotton rat model.

## Methods

### Animals and ethics statement

Six to seven week old cotton rats were obtained from Envigo, Somerset, N.J., USA. All animal experiments were reviewed and approved by Institutional Animal Care and Use Committee of Health Canada and were conducted in accordance with Institutional Animal Care and Use Committee of Health Canada guidelines and regulations.

### Cells, viruses and vaccines

293 T (ATCC: CRL-3216) were grown in Dulbecco’s Modified Eagle Medium (DMEM) supplemented with sodium bicarbonate, HEPES, Penicillin, Streptomycin, and 10% FBS.

Primary bone marrow derived dendritic cells from C57BL/6 mice were cultured in media from manufacturer (Cell Biologics) supplemented with 10% FBS, 2-mercaptoethanol, L-Glutamine, Penicillin and Streptomycin. HEp-2 (ATCC: CCL-23) were grown in DMEM supplemented with sodium bicarbonate, Glutamax, HEPES, Penicillin, Streptomycin, and 10% FBS.

RSV-A2 (ATCC: VR-1540) was grown in HEp-2 cells according to supplier’s instructions and sucrose-purified for animal studies. FI-RSV was prepared with the RSV-A2 strain in HEp-2 cells as described elsewhere^60^. FI-Mock was made with uninfected HEp-2 cells using the same procedure as FI-RSV.

### Isolation and sequence determination of cotton rat PD-1 cDNA

Total RNA was isolated and 3′ RACE was conducted as previously described^49^. Briefly, spleens from naïve cotton rats were removed aseptically and frozen. An eighth of the spleens were cut and homogenized with a TissueLyser II (Qiagen). Total RNA was extracted using the RNeasy Mini kit (Qiagen) with on-column DNase digestion according to the manufacturer’s instructions. The 3′ RACE system (Life Technologies) was then used to amplify the 3′ portion of the cotton rat PD-1 from the total RNA according to the user’s manual. Following first strand cDNA synthesis using an oligo dT adapter primer, the 3′ portion of the cotton rat PD-1 mRNA was PCR amplified using the abridged universal amplification primer and consensus sequences derived gene specific primer (5′–GGAGTCCGGTTCTGTGTACCT–3′) at an annealing temperature at 55 °C. The gene specific primer was determined by aligning the PD-1 sequences of *Microtus ochragaster* (NCBI Reference Sequence: XP_005361412.1), *Cricetulus griseus* (NCBI Reference Sequence: XP_003499314.1), *Mus musculus* (NCBI Reference Sequence: NP_032824.1) and *Rattus norvegicus* (NCBI Reference Sequence: XP_017451871.1). The PCR products obtained were run on a DNA gel and excised using a Qiagen QIAquick Gel Extraction kit as per manufacturer’s procedure. All amplified fragments were sequenced with BigDye Terminator v.3.1 Cycle Sequencing kit (ThermoFisher) using a 3130xl Genetic Analyzer (Applied Biosystems) following amplification in a PTC-200 thermal cycler (MJ Research). Raw sequencing data was edited using 3130xl Genetic Analyzer Data Collection Software v3.0 (Thermo Fisher), and then imported into GeneCodes Sequencher v4.6.1 sequencing analysis software for further editing. The final sequenced contigs were then imported to NCBI BLAST (https://blast.ncbi.nlm.nih.gov/Blast.cgi) to confirm the identity. Reverse primers were designed as fragments of the gene were sequenced until the gene encoding the complete PD-1 protein (determined by aligning against other species) was found.

### Sequence and phylogenetic analysis

Putative functional domains, intracellular and extracellular domains, and ligand binding sites were determined using a standard protein BLAST (https://blast.ncbi.nlm.nih.gov). Sequences with significant alignments were imported into Geneious Pro version 5.6.7 (Auckland, New Zealand) for phylogenetic analysis. Multiple alignment was conducted using the Clustal Omega tool from EMBL-EBI.

### crPD-1 gene synthesis, protein expression and purification

After confirmation of the full mRNA sequence, the gene was synthesized and cloned into a pcDNA3.1(+) vector (GenScript). The synthesized gene began with a kozak sequence (5′-GCCGCCACC-3′) followed by a 23-amino acid secretion signal (MLLAVLYCLLWSFQTSAGHFPRA) from the human tyrosinase signal peptide as previously shown^61^. Following the secretion signal, ten histidine residues were added to facilitate protein purification, followed by the complete 1885 bp crPD-1. Rat-codon optimized sequences were used for gene synthesis.

293T cells were transiently transfected with the pcDNA vector containing the recombinant crPD-1 for 5 hours with Lipofectamine® 2000 (Invitrogen). After the 5-hour incubation, the lipofectamine-DNA containing media was removed, growth media was added and cells were incubated overnight at 37 °C. The next day, cells were washed, scraped and lysed with a standard RIPA cell lysis buffer. Following sonication of the lysed cells, the samples were his-tag purified using a His Mag Sepharose^TM^ excel kit (GE Healthcare) according to manufacturer’s instructions. The samples were then dialyzed in PBS for functional studies.

### Western blot, mass spectrometry and immunofluorescence

Samples resulting from his-tag purification were used for western blotting and mass spectrometry for confirmation of protein expression. A recombinant mouse PD-1 protein with a his-tag at the C-terminus (Abcam) was used alongside. Western blotting was performed using 4 to 15% TGX gel and Tris/Glycine/SDS running buffer (Bio-Rad Laboratories Inc.). The protein samples were then transferred to PVDF membranes (Millipore) and detected with mouse tetra-His antibody (Qiagen) and goat anti-mouse IRDye-800CW (LiCor). Membranes were visualized using the Odyssey system (LiCor).

Mass spectrometry was performed after the samples were cysteine-reduced, alkylated, and then digested on filter with trypsin. Resulting peptides were injected onto an Easy-nLC 1000 for reversed phase separation and analyzed with an Orbitrap Fusion Tribrid Mass Spectrometer operating in DDA mode with HCD fragmentation. The data was processed with Proteome Discoverer 2.2 software searching databases of rat proteins, common laboratory contaminants, and crPD-1.

Immunofluorescence was used for further confirmation of protein expression. 293 T cells were seeded at a density of 30,000 per well in growth media in a 96-well flat clear bottom black plates. Next day, the cells were transfected with 1 µg crPD-1 in pcDNA vector for 24 hours. On the following day, transfected cells were fixed with cold cytofix/cytoperm (BD Biosciences). After blocking with 3% IgG-free BSA diluted in wash buffer (1x PBS with 0.1% Tween 20) for 1 hour at 37 °C, the cells were stained with a mixture of unconjugated rabbit anti-human/mouse/rat PD-1 antibody (Abcam) and mouse tetra-His antibody (Qiagen) for 1 hour at 37 °C. Then, a mixture of Alexa Fluor 555-conjugated anti-mouse IgG (Invitrogen) and Cy2-conjugated anti-rabbit IgG (Jackson Immunoresearch) was added for 1 hour at 37 °C. The cells were imaged using the EVOS FL microscope: Alexa Fluor 555 in the RFP and Cy2 in the GFP channel.

### Flow cytometry

Dendritic cells were seeded at 300,000 per well in growth media in a 96-well round bottom plate. Seeded cells were incubated with 30 µg/ml purified and dialyzed crPD-1 or recombinant mouse PD-1 (Abcam) for 4 hours at 37 °C. Following the incubation, the plate was centrifuged, supernatant was removed and the cells were stained for flow cytometry analysis. Cell suspensions were washed with PBS and first, stained with Fixable Viability Dye eFluor® 506 (eBioscience) for 30 min, then, with purified anti-mouse CD16/CD32 (eBioscience) as a Fc block for 5 min. Next, cells were washed with FACS wash buffer (PBS with 1% BSA and 0.05% sodium azide) and either stained with PE-conjugated anti-human/mouse PD-L1 antibody (CD274; eBioscience, clone MIH1) or rabbit anti-mouse PD-1 (Abcam, clone EPR20665) along with a PE-conjugated anti-rabbit IgG. Stained samples were run on the same day on a BD LSRFortessa flow cytometer. Data analysis was completed using FACSDiva version 8.0.1.

### Quantitation of cytokines

Dendritic cells were seeded in growth media containing 0.02 µg/ml LPS. The cells were incubated with or without 20 µg/ml crPD-1 or recombinant mouse PD-1 (Abcam) for 24 hours at 37  °C. The next day, the plates were centrifuged and supernatant was collected for cytokine quantitation using ELISA. Mouse DuoSet® ELISA Kits (R&D Systems) for IL-6 and TNF-α were used as per manufacturer’s procedure. The absorbance was read on a BioTek Synergy 2 plate reader.

### Animal studies

On day 0 and day 21, 6 to 7-week old cotton rats were intramuscularly vaccinated with 1 × 10^6^ PFU FI-RSV, FI-Mock or PBS buffer. The RSV group was intranasally vaccinated at 1 × 10^6^ PFU. On day 49, all animals were challenged intranasally with 1 × 10^6^ PFU of RSV-A2. Five days post-challenge, the animals were euthanized. The lungs were removed and one lobe was used for virus titration while the other lobe was fixed in 10% neutral buffered formalin (Sigma) under 25 cm of water pressure. Lungs for RNA isolation were snap frozen in liquid nitrogen.

### Lung viral titration

Lungs were removed 5 days post RSV challenge and tittered as described elsewhere^60^. Briefly, half the lungs were collected in serum free RPMI media and weighed prior to mechanical homogenization. The homogenates were clarified using centrifugation and the supernatants were serially diluted and incubated on HEp-2 cells for 2 hours at 37 °C. A 1:1 overlay of 2x DMEM media and 0.8% agarose was added. Following 6 days of incubation, the overlay was removed and the cell monolayer was stained with crystal violet before counting plaques. Results are expressed as PFU/g lung tissue.

### Lung histology

Half the lung fixed in 10% formalin 5 days following RSV challenge were trimmed, processed and embedded into paraffin blocks. Five-micron H&E stained slides were made for evaluation by a certified veterinary pathologist who was blinded to the experimental design. Each sample was assessed for peribronchiolitis and alveolitis.

### Real-Time quantitative PCR

Frozen lungs from PBS, RSV, FI-RSV and FI-Mock immunized and RSV challenged cotton rats were cut, homogenized and total RNA was extracted as described above for spleens. Superscript III First Strand Synthesis System (Invitrogen) was used to generate cDNA according to manufacturer’s instructions. The cDNA was then used for quantitative PCR using TaqMan® Fast Advanced Master Mix (Applied Biosystems) as per the manufacturer’s procedure. A forward primer (5′-CACTGTAACCTATGACCTCTGG-3′), a reverse primer (5′-CCTTTTCCCTCTTTTGATGCTG-3′) and a TaqMan® probe (5′-TTGCCTCTCCCTACTCTTCCCCT-3′) with a MGBNFQ 3′ quencher and 6FAM 5′dye were designed to target crPD-1. β-actin was used as the reference gene and was targeted using a primer-probe set described elsewhere^62^. Quantitative real-time PCR was conducted on an ABI Prism 7500 Fast Sequence detection system (Applied Biosystems) and C_T_ values were obtained. Fold change over control immunization groups was calculated using the ΔC_T_ method using β-actin as the reference gene^63^.

### Immunohistochemistry

Half the lungs from PBS, RSV, FI-RSV and FI-Mock immunized cotton rats were fixed in 10% formalin 5 days following RSV challenge, trimmed, processed and embedded into paraffin blocks. Charged slides were made from the blocks and used for immunostaining as previously described^64,65^ with some modifications. Following 10 min antigen retrieval by boiling in 1 mM Tris/EDTA buffer (pH = 9.0) in a pressure cooker, sections were blocked with Protein Block (X0909, DAKO) for 2 h, and incubated overnight at 4 °C with rabbit anti-mouse PD-1 (Abcam, clone EPR20665). Then, the samples were incubated with a secondary anti-rabbit IgG followed by addition of diaminobenzidine (DAB) substrate.

For each section, 8–10 fields of view were counted at 20X magnification. Counting was performed using Northern Eclipse software and thresholds were set using negative controls. Fold change of percent PD-1 positive cells in the immunized groups over unimmunized control groups is presented.

### Statistical analysis

Analysis was conducted using unpaired Student’s t-test, one-way ANOVA where appropriate. Bonferroni posttest was used to adjust for multiple comparisons between different test groups. Tests were done at a 5% significance level. All statistical analyses were performed using GraphPad Prism 7 software.

## Supplementary information

Supplementary Data

## Data Availability

The datasets generated during and/or analyzed during the current study are available from the corresponding author on reasonable request. The mRNA and amino acid sequence of crPD-1 can be found in GenBank: Accession # MK040464.
