# Supplementary material for: PD-1 of Sigmodon hispidus: Gene identification, characterization and preliminary evaluation of expression in inactivated RSV vaccine-induced enhanced respiratory disease
Source: Sci Rep. 2019 Aug 12;9:11638. doi: 10.1038/s41598-019-48225-x (PMC6690999; doi:10.1038/s41598-019-48225-x)
Supplement: Supplementary file 1 — Supplementary Data [file 41598_2019_48225_MOESM1_ESM.docx]

**Supplementary Information**

**PD-1 of *Sigmodon hispidus*: Gene identification, characterization and preliminary evaluation of expression in inactivated RSV vaccine-induced enhanced respiratory disease**

Abenaya Muralidharan^1,3^, Louise Larocque^1^, Marsha Russell^1^, Marybeth Creskey^1^, Changgui Li^2^, Wangxue Chen^4^, Gary Van Domselaar^5^, Jingxin Cao^5^, Terry Cyr^1^, Michael Rosu-Myles^1,3^, Lisheng Wang^3^, Xuguang Li^1,3,*^

^1^Centre for Biologics Evaluation, Biologics and Genetic Therapies Directorate, HPFB, Health Canada and WHO Collaborating Center for Standardization and Evaluation of Biologicals, Ottawa, ON, Canada.

^2^National Institute for Food and Drug Control and WHO Collaborating Center for Standardization and Evaluation of Biologicals, Beijing, China

^3^Department of Biochemistry, Microbiology and Immunology, Faculty of Medicine, University of Ottawa, Ottawa, ON, Canada

^4^Human Therapeutics Portfolio, National Research Council of Canada, Ottawa, ON, Canada

^5^National Microbiology Laboratory, Public Health Agency of Canada, Winnipeg, MB, Canada

^*^Corresponding author


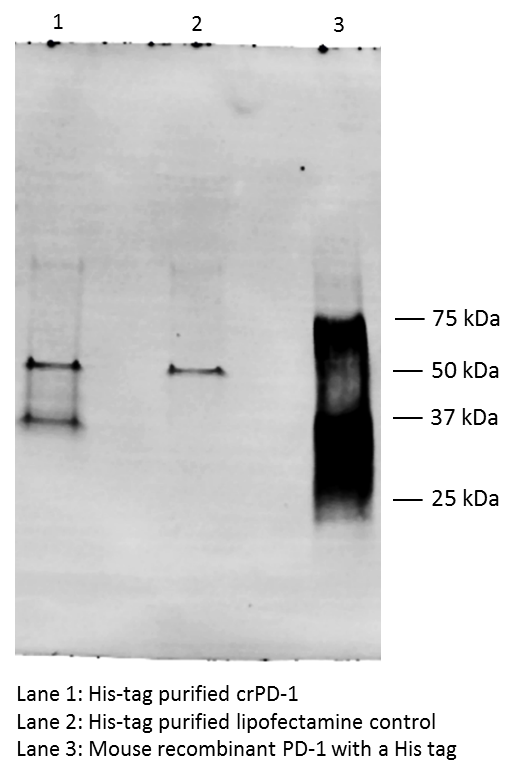


**Supplementary Fig. S1: Cotton rat PD-1 protein expression.** crPD-1 gene also encoding rat codon optimized secretion signal and ten histidine residues at the 5’-end was synthesized and cloned into pcDNA3.1(+) vector. 293T cells were then transfected for 24 hours, the lysate was collected and His-tag purified. Protein expression was confirmed with western blot using a mouse anti-histidine antibody. The expected size of crPD-1 is 36.4 kDa and was only observed in His-tag purified crPD-1.
